# Supplementary material for: Orthogonal-view Microscope for the Biomechanics Investigations of Aquatic Organisms
Source: ArXiv. 2023 Jul 24:arXiv:2307.13079v1. Preprint. [Version 1] (PMC10402206)
Supplement: 1 — Supplementary Figure −1: Preliminary results demonstrating fluorescent imaging. Supplementary Figure −2: GLUBscope - case studies [file NIHPP2307.13079V1-supplement-1.pdf]

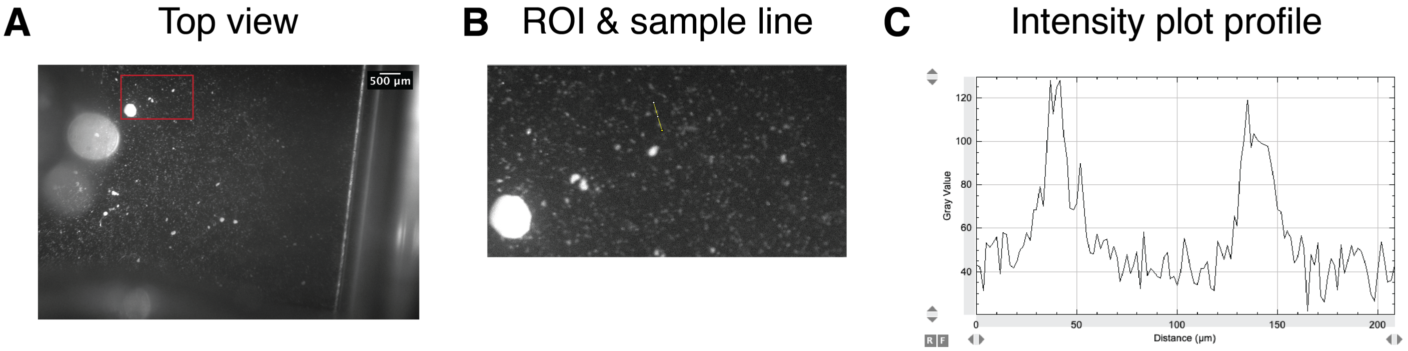

Figure 6: **Supplementary Figure-1: fluorescent imaging.** (A) Green fluorescent beads (5 microns) were mixed in DIY water and vortexed inside a cuvette (3.5 mL volume and path length 1 cm). An off-the-shelf Green LED was used to illuminate the sample. (B) A region of interest (ROI) and a sample line for intensity measurements were selected. (C) The intensity profile along the sample line indicating the bead clusters. The signal to noise could be improved by using brighter excitation light source and reducing background .

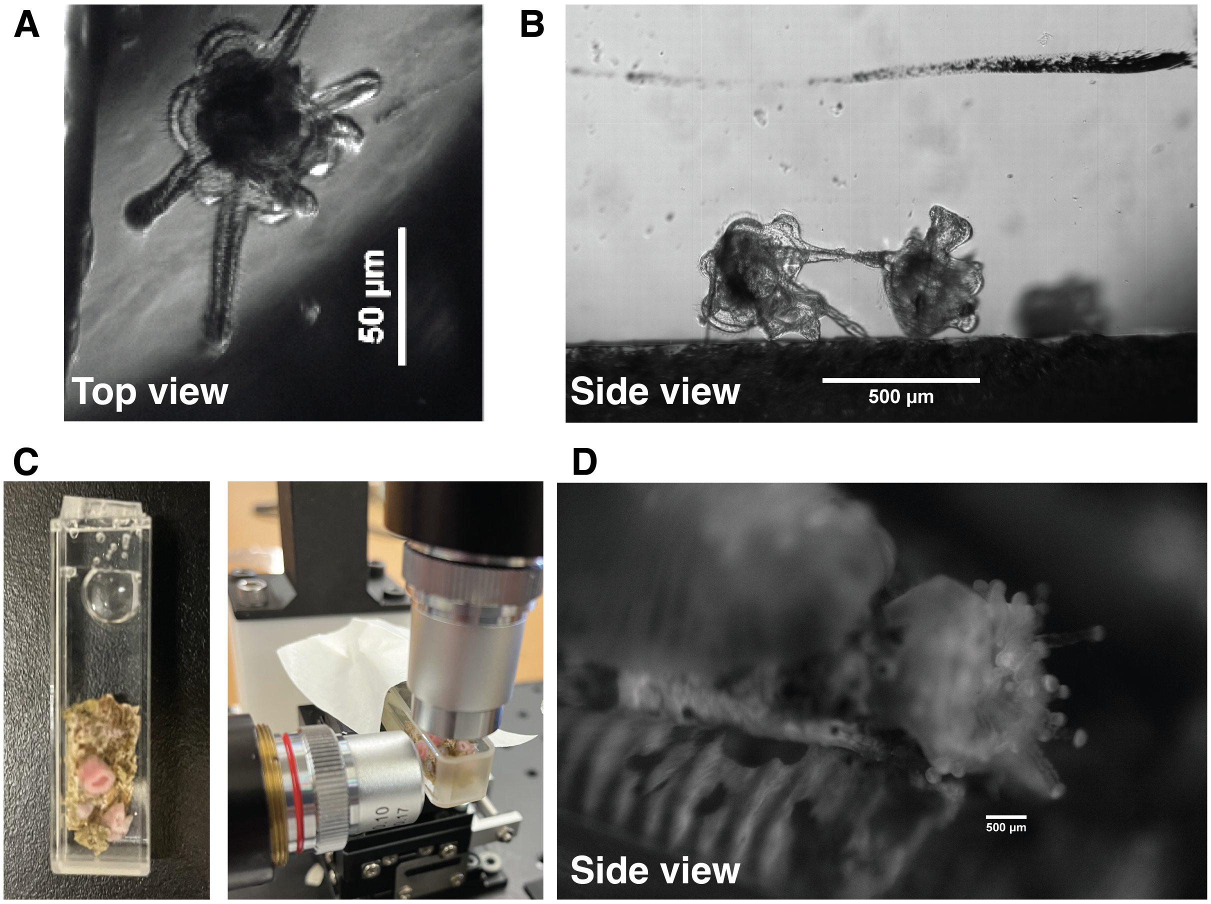

Figure 7: **Supplementary Figure -2: GLUBscope - case studies**. (A-B) top and side views of sand dollar larvae (C) Sea anemone attached to a rock inside the sample holder and on the stage. (D) Sea anemone imaged from the side view with GLUBscope.
